# New association of milk thistle and artichoke extracts enhances egg quality in caged-laying hens

**DOI:** 10.3389/fvets.2025.1702920

**Published:** 2025-10-27

**Authors:** Valentina Serra, Francesca Leone, Valeria Harper, Lorenzo Fiorini, Francesca Del Zozzo, Thibaut Chabrillat, Claire Carlu, Ivonne Laura Archetti, Grazia Pastorelli, Doriana Eurosia Angela Tedesco, Alessandro Guerrini

**Affiliations:** ^1^Department of Veterinary Medicine and Animal Science, University of Milan, Lodi, Italy; ^2^Department of Environmental Science and Policy, University of Milan, Milan, Italy; ^3^Independent Researcher, Imola, Italy; ^4^Chemifarma S.p.a., Forlì-Cesena, Italy; ^5^Phytosynthese, Mozac, France; ^6^Istituto Zooprofilattico Sperimentale della Lombardia e dell'Emilia Romagna "Bruno Ubertini" (IZSLER), Brescia, Italy

**Keywords:** bioactive compounds, hens’ health, egg-quality indices, peak of production, antioxidants, nutraceutical properties

## Abstract

**Introduction:**

Egg quality is crucial to productivity and laying hens’ health. However, hens’ aging, oxidative stress, and metabolic disorders (e.g., liver steatosis) can impair egg production and quality during the production cycle. Nutritional interventions may help preserve productivity under these conditions. Among plant extracts, milk thistle (*Silybum marianum* L.) and artichoke (*Cynara scolymus* L.) are noteworthy for their bioactive compounds with hepatoprotective and antioxidant properties. This study evaluated the effectiveness of a combined extract of milk thistle and artichoke (PHYTO-LAYER™), standardized in silibinin (2.4 g/L) and chlorogenic acid (2.2 g/L), in maintaining or improving egg quality, lipid oxidation, and antioxidant capacity in caged-laying hens exposed to hepatic and metabolic stress.

**Methods:**

A total of 792 Lohmann LSL-White hens (41 weeks old) were randomly assigned to two groups, control and treated (396 hens per group). The treated group received the products via drinking water at a dose of 1 mL/L, intermittently for 7 weeks (7 consecutive days every 2 weeks). Sampling occurred at five time points (T0–T5). At T0, T3, and T5, 60 eggs per group were collected for quality indices evaluation, while 13 hens per group were sampled for serum biochemical investigations.

**Results:**

PHYTO-LAYER™ improved egg, yolk, and albumen weight (*p* < 0.000), eggshell thickness (*p* < 0.000), and the total polyphenol content (*p* < 0.026), with an enhancement of yolk antioxidant capacity (*p* < 0.024). However, the Haugh unit of treated eggs was reduced (*p* < 0.000).

**Discussion:**

Egg quality often deteriorates during late production stages due to oxidative stress and hens’ aging. Given the antioxidant potential of silibinin and chlorogenic acid, their combined intermittent administration supports and maintains the egg quality in caged-laying hens exposed to metabolic stress and after the peak of production. However, further studies could be of interest to verify whether similar changes in egg-quality indices are observed with other phytoextract administration protocols, such as continuous administration, and at different administered doses.

## 1 Introduction

Egg quality includes a wide range of indices, such as egg weight, thickness and strength, and albumen weight and its Haugh unit, representing a pivotal aspect of the laying hen industry, directly impacting productivity and overall hens’ health (1). These indices, associated with other parameters such as egg nutritional composition, antioxidant, and nutraceutical characteristics, are crucial indicators to define the egg’s physical properties, nutritional content, and overall integrity (2). During the egg production cycle, and with hens’ aging, hens experience a decline in egg-laying performance, which can be accelerated by strong metabolic stress or common disease episodes (e.g., liver disease, osteoporosis), oxidative stress, and immune imbalance (3–6). Among these, liver steatosis and fatty liver haemorrhagic syndrome (FLHS) are the most common multifactorial metabolic disorders, which occur especially in caged hens during and after the peak of production (7, 8). Therefore, it is essential to explore new ways to mitigate these health issues, ensure the continuity of egg production, and maintain the stability of egg quality during the production cycle.

Although no unique and consolidated strategies are currently available to counteract liver steatosis or FLHS in laying hens, plant-based feed additives derived from herbal extracts represent a promising option. Many studies have shown that phytoactive compounds supplementation in aged (~60 weeks of age) or post-peak (~26–28 weeks of age) healthy laying hens improves egg quality and lipid metabolism and alleviates oxidative stress (9–18). Interesting results on egg quality were observed, for example, in laying hens at the peak of production (26 weeks of age) supplemented with a combination of phytoextracts (PHYTO AX’CELL™) at a dose of 1 mL/L, administered intermittently via drinking water, containing *Curcuma longa* L. (Turmeric), *Gaultheria procumbens* (American Wintergreen), and green propolis (19).

Among plant-derived extracts, milk thistle (*Silybum marianum* L.) and artichoke (*Cynara cardunculus scolymus* L.) have gained attention and practical application in poultry, due to their high content of bioactive compounds. In particular, milk thistle, the main source of silymarin (primarily silibinin—SIL), a flavonolignan complex known for its medicinal properties in the treatment of liver disorders, exhibits marked hepatoprotective and antioxidant activities (17), attributed to its ability to inhibit free radical production and to stimulate detoxification processes (20). Despite this, the effect of its main bioactive compound SIL has rarely been investigated in laying hens after the peak of production or late-laying (21). Artichoke, known for its choleretic, diuretic, cholesterol-reducing, and hepatoprotective action, is a good source of phenolic and antioxidant compounds, such as cynarin and chlorogenic acid (CGA) (22). The CGA, a bioactive polyphenol that contains five active hydroxyl groups and one carboxyl group, has been identified as a powerful antioxidant that removes reactive oxygen species (ROS) with protective effects against oxidative damage (23). Artichoke, a source of CGA, improves the poultry performance and quality products (e.g., improvement of feed conversion ratio, egg mass, egg-quality characteristics, reduction of the fat ratio in the liver of laying hens) (24).

Given the known properties of SIL and CGA, it is plausible that their administration could influence the antioxidant capacity or polyphenol content of eggs. In this regard, it has been demonstrated that supplementation with polyphenols derived from other plant sources (e.g., Green tea) can enhance yolk antioxidant capacity (25). To the best of our knowledge, the individual and synergistic effects of extracts from these two medicinal plants on egg-quality parameters and the antioxidant capacity of eggs produced by hens with liver disorders have not yet been clearly investigated. Previous studies showed that the administration of a combination of SIL + CGA standardized extract (PHYTO-LAYER™), administered intermittently at a dose of 1 mL/L to 41-week-old laying hens affected by liver disorders (liver steatosis and FLHS), improved liver function by reducing alanine-aminotransferase (ALT) enzyme activity, ameliorated the severity of liver steatosis, and improved some blood indices (e.g., red blood cells, hemoglobin and hematocrit) after the peak of production (26). These findings, also associated with an improvement in total serum antioxidant capacity, suggested that eggs produced by treated hens could have different qualitative characteristics compared to the control ones.

Therefore, to advance and deepen the study, considering that egg quality may deteriorate in caged-laying hens affected by liver disorders and prolonged high production, the same experimental hens were evaluated to assess whether administration of the SIL + CGA complex could help maintain or improve their egg quality.

## 2 Materials and methods

### 2.1 Ethics statement

The data reported and discussed in this manuscript are part of an experimental study evaluated and approved by the Ethical Committee of the University of Milan and the Italian Ministry of Health, under protocol no. 766/2023-PR.

### 2.2 Farm characteristics and management

A commercial laying hen farm was selected for the field study. The farm included two separate nearby sheds, equally oriented, where the hens were raised in enriched cages (25 to 26 hens/cage). The cage battery in each shed consisted of four rows of five-decker cages, with single cages placed on the right and left side of each row and deck. Each cage was equipped with a common nest, scratching area, linear feeder, six nipple-type drinkers positioned along the longer lateral wall of each cage, and three plastic perches (20 cm/hen). All animals were maintained under identical management and commercial conditions and exposed to a standard lighting program (16 L:8D, 20 lux), with NH_3_ and CO_2_ levels maintained at <20 ppm and <3,000 ppm, respectively, temperatures between 20 °C and 26 °C, and ~60% relative humidity throughout the trial. The water and feed distribution lines were separated and independent between the two sheds. To ensure a homogeneous, continuous, and ad libitum water administration containing the dose of supplements per liter, the automatic Dosatron impulse-pump system (SmartDosing© D25AL5N, Dosatron, USA) was used.

### 2.3 Experimental design and treatment

**Figure 1:**
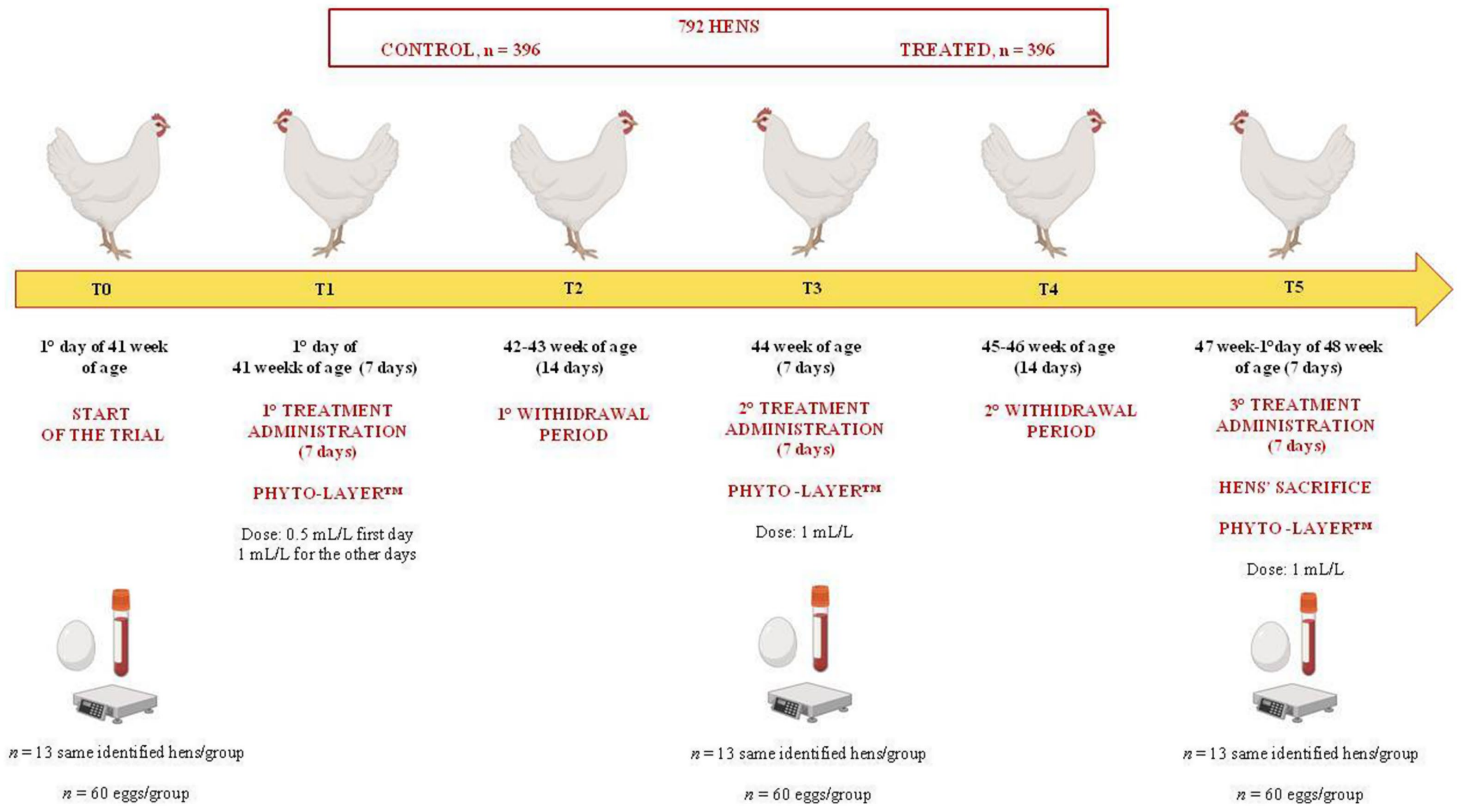
Experimental design of the study.

The experimental design is illustrated in Figure 1. In a 7-week trial, a total of 792 Lohmann LSL-White laying hens (1.667 ± 0.13 kg/body weight-BW), non-beak-trimmed, and from the same commercial flock, upon reaching the 41st week of age and egg production rate (above 90%), were randomly chosen and assigned to two separate and replicated experimental groups, control and treatment (396 hens each). In each shed, the 396 selected hens represented a single separate group, control or treated. Each experimental group allotted to each shed had 15 replicate cages (25–26 hens each) randomly identified and alternated among the different rows and decker shed cages of the shed, both on the right and left side of the battery system. For each treatment group, 13 hens (1 hen per cage, established based on preliminary statistical power analysis; control, *n* = 13; treated, *n* = 13) were randomly identified for blood sampling and were evaluated at each time point of the trial.

### 2.4 Complementary feed tested

The study was designed to evaluate the intermittent administration via drinking water of an authorized complementary feed (PHYTO-LAYER™ product, provided by Phytosynthese, France), on egg-quality indices. The commercial product, administered at a dose of 1 mL/L, was based on an association of two standardized extracts: milk thistle (*Silybum marianum* L., Council of Europe—CoE: 551) seeds extract (water-acetone, 70:30 v/v) and artichoke (*Cynara cardunculus scolymus* L. CoE: 565) leaves water extract (27), expressed as content of SIL (2.4 g/L) and CGA (2.2 g/L), respectively.

The treatment was administered according to five experimental time points (T0-T5) (Figure 1), as follows:

- T0: 1st day of the start of the 41st week, before the start of the treatment.
- T1: 41st week (7 days), first treatment administration (0.5 mL/L only the first day of the treatment to accustom the animals to any changes in the water taste and not to compromise the water intake and egg production).
- T2: 42nd to 43rd week (14 days), first withdrawal period.
- T3: 44th week (7 days), second treatment administration.
- T4: 45th to 46th week (14 days), second withdrawal period.
- T5: 47th week to the first day of the 48th week of age until the end of the trial (7 days), third treatment administration.

At T5, the 13 identified hens per group were sacrificed by manual cervical dislocation, for the latest blood sampling and liver anatomopathological and histological investigations conducted in the first phase of the same study. The preliminary results reported by Guerrini and colleagues (26) showed that the experimental hens’ groups were affected by liver disorders, including cases of liver steatosis and FLHS, evaluated histologically. Treated hens showed a reduction of serum ALT enzyme activity (treated: 8.00 ± 2.16 U/L at T0 vs. 5.92 ± 1.38 U/L at T5; control: 8.91 ± 2.57 U/L at T0 vs. 7.00 ± 2.19 U/L at T5), a lower liver weight (39.91 ± 3.62 g vs. 44.61 ± 6.97 g in treated and control group, respectively), and an enhancement of serum antioxidant capacity (T-OAC; treated: 1.35 ± 0.75 mM Trolox eq. at T0 vs. 2.82 ± 0.48 mM Trolox eq. at T5; control: 1.29 ± 0.68 mM Trolox eq. at T0 vs. 1.94 ± 0.89 mM Trolox eq. at T5) (26). During the 7-week experimental period, all hens were fed ad libitum with the same commercial diet to meet the requirements for Lohmann LSL-White laying hens. The composition and nutrient levels of the basal diet are reported in Table 1.

**Table 1:** Composition and nutrient levels of the basal diet supplied to laying hens during the trial (% as-fed basis).

Items	Content (% as-fed basis)
Basal ingredient	
Corn	31.03
Wheat	22.90
Soybean meal (46.5% crude protein, CP)	14.14
Sunflower	10.00
Wheat flour	8.00
Calcium	5.40
Calcium carbonate (0.5 mm)	3.60
Soybean oil	2.00
Dicalcium phosphate	1.00
Canthaxanthin+lutein	0.80
Mineral and vitamins premix^1^	0.39
Lysine	0.16
Sodium sulfate	0.15
NaCl	0.15
Methionine	0.13
Choline	0.06
Digestive promoters—phytase	0.05
Threonine	0.04
Total	100
Nutrient level	
Metabolisable energy (ME, kcal/kg)	2.715,10
Crude protein	16.50
Ether extract	4.14
Crude fibre	4.09
Ash	12.51
Ca	3.83
P	0.62
Available P	0.38
L-lysine	0.89
Available lysine	0.75
DL-methionine + cysteine	0.70
Available methionine + cysteine	0.60

1 Mineral, vitamins, and organic acids premix supplied per kg of diet: vitamin A, 3,334,000 IU; vitamin D3, 100,000 IU; vitamin E, 834 mg; vitamin K3, 1,666 mg; vitamin B1, 3,334 mg; vitamin B2, 1,666 mg; vitamin B6, 8 mg; vitamin B12, 34 mg; niacinamide, 11,680 mg; folic acid, 334 mg; calcium D-pantothenate, 3,334 mg; Fe, 11,680 mg; Mn, 41,680 mg; Zn, 20,000 mg; Cu, 2,000 mg; iodine, 166 mg; Se, 150 mg.

### 2.5 Blood sampling

Blood samples (2 mL) were collected at T0 and at the end of the first withdrawal period (T3) from the same identified 13 hens per group from the brachial vein, using a sterile syringe with a 23 G-0.60 mm needle, while at the end of the trial (T5), blood samples (10 mL) were collected via jugular vein transection. After each collection, the blood was kept in serum glass tubes without anticoagulant (BD Vacutainer, Becton Dickinson, Milan, Italy) to obtain the serum after centrifugation at 2,500 x g at 4 °C for 15 min, and subsequently stored in 5-mL plastic vials at −20 °C until analysis of serum biochemical indices. The analyses were performed in the laboratory of the Istituto Zooprofilattico Sperimentale della Lombardia e dell’Emilia-Romagna (IZSLER).

### 2.6 Serum biochemical analysis

Serum biochemistry indices, including total proteins (TP, g/L), albumin (Alb, g/L), globulins (Glb, g/L) and their ratio (Alb/Glb), calcium (Ca, mmol/L), phosphorus (P, mmol/L), and magnesium (Mg, mmol/L), were measured spectrophotometrically using a biochemical multi-analyzer ILab 650 (Instrumentation Laboratory, Werfen Company, Milan, Italy), and kits provided by the same manufacturer.

### 2.7 Egg-quality characteristics

From each experimental group, 60 eggs were randomly collected for the evaluation of quality indices at T0, at the end of the first withdrawal period (T3), and at the end of the trial (T5) (Figure 1). A sample of 60 eggs per experimental group provides sufficient observations to estimate means and variances with good precision while balancing statistical robustness with practical feasibility (28–32). Of the 60 eggs for each experimental group, 30 eggs were entirely weighed (grams, g), and the yolk and shell weights were assessed using an electric balance, while the albumen weight was calculated as the difference between the whole egg weight, yolk, and eggshell weight. Subsequently, the eggshell thickness was measured using a digital caliper (0.01 mm) at three different points at the equatorial level and expressed as a mean of three measurements (mm) ± standard deviation (SD).

For the other 30 eggs per group, the breaking strength was measured using the Egg Force Reader™, which applies a force to the egg’s sharp pole to cause cracks or breakages in the shell. The results were expressed in Newtons (N). The height of the albumen was measured using a tripod micrometer at three different points near the yolk, and the Haugh unit (HU) was calculated using the following formula: HU = 100 * Log10 (h – 1.7w 0.37 + 7.6), where: h = observed height of the albumen in millimeters (mm); w = weight of egg in grams (g). Additionally, the yolk color was evaluated using the Digital YolkFan™ Colorimeter (DSM-Firmenich, Denmark).

### 2.8 Egg yolks’ lipid oxidation

From each experimental group cage, 10 eggs were randomly collected to determine lipid oxidative status and antioxidant activity in yolk samples, at three different time points (T0, T3, and T5), for a total of 60 eggs. Eggs were carefully broken to recover the yolks, which were immediately frozen at −80 °C until analysis. The yolk samples were evaluated for lipid oxidation by determining the concentration of thiobarbituric acid-reactive substances (TBARS) following the protocol described in Pasri and colleagues (33). For this protocol, 2 g of each egg yolk was weighed in a 15-mL tube; then 6 mL of deionized water and 34 μL of 7.2% butylated hydroxy toluene (BHT; Cayman Chemical, Ann Arbor, MI, USA; Cat. no. 89910) were added and mixed using an Ultra-Turrax homogeneiser (model T-25 basic, IKA-Labortechnik, Janke & Kunkel, GMBH, Stuttgart, Germany). Then, 2 mL of the egg yolk emulsion was mixed with 4 mL of a solution made up of 20 mM thiobarbituric acid (TBA; Sigma-Aldrich, St. Louis, MO, USA; Cat. no. T5500) and 15% trichloroacetic acid (TCA; Sigma-Aldrich, St. Louis, MO, USA; Cat. no. T6399) and boiled at 95 °C for 20 min. The mixture was then centrifuged at 5,000 x g for 10 min at room temperature, and 200 μL of the supernatant was pipetted in triplicate into wells of a 96-well microtiter plate (Nunc™ MicroWell™, ThermoFisher Scientific™).

Absorbance values were measured at 532 nm using a spectrophotometer (Epoch Biotek, Agilent Technologies Inc., Santa Clara, CA, USA). A malondialdehyde (MDA; Cayman Chemical, Ann Arbor, MI, USA; Cat. no. 10009202) standard solution (125 μM) was serially diluted to prepare a calibration curve ranging from 0 to 50 μM. A standard curve was generated for each analysis set by plotting absorbance at 532 nm against MDA concentration. To correct for possible matrix interferences, sample blanks without TBA reagent were run in parallel, and their absorbance values were subtracted from the corresponding TBARS measurements. The results were expressed as μM MDA.

### 2.9 Egg yolks, total phenolic content, and antioxidant activity

The total phenolic content (TPC) in yolk samples was determined through the Folin–Ciocalteu (FC) assay according to Attard and co-workers (34). Before performing the assay, 2 g each of egg yolk was mixed with 4 mL of 99% ethanol using an Ultra-Turrax homogeneiser, model T-25 basic, IKA-Labortechnik, (Janke & Kunkel, GmbH, Stuttgart, Germany) and then centrifuged at 12,000 g at 4 °C for 10 min. The supernatant (ethanol extract) was used to evaluate the TPC following the microtiter plate protocol. Briefly, 10 μL of each ethanol extract or gallic acid dilution was pipetted in triplicate into wells of a 96-well microtiter plate (Nunc™ MicroWell™, ThermoFisher Scientific™). After adding 100 μL of 10% FC reagent (Sigma-Aldrich, St. Louis, MO, USA; Cat. No. F9252) and 80 μL of 1 M sodium carbonate (Na_2_CO_3_) (Carlo Erba Reagents, Milano, Italy; Cat. No. 479306) to each well, the plate was allowed to incubate in the dark for 20 min at room temperature, and absorbance was measured at 630 nm on a spectrophotometer (Epoch Biotek, Agilent Technologies Inc., Santa Clara, CA, USA). Gallic acid (Sigma-Aldrich, St. Louis, MO, USA; Cat. No. 398225) standard solution (960 μg/mL) was serially diluted up to 60 μg/mL to construct the calibration curve. TPC was expressed as mg gallic acid equivalents (GAE) per gram of sample (mg GAE/g). The ethanol extract of egg yolk samples was used to determine the antioxidant activity through the 2′-azinobis-(3-ethylbenzothiazoline-6-sulphonic acid) (ABTS) assay following the protocol described by Re and colleagues (35), with some modifications. In detail, ABTS (Sigma-Aldrich, St. Louis, MO, USA; Cat. No. A1888) was dissolved in distilled water to obtain the ABTS stock solution (7 mM). Subsequently, the ABTS radical cation (ABTS^•+^) was produced by reacting ABTS stock solution with 2.45 mM potassium persulfate (Sigma-Aldrich, St. Louis, MO, USA; Cat. No. 216224) and allowing the mixture to stand away from light at room temperature for 12–16 h before use. Trolox (6-hydroxy-2,5,7,8-tetramethoxychroman-2-carboxylic acid; Sigma-Aldrich, St. Louis, MO, USA; Cat. No. 238813) standard solution (2 mM) was diluted up to 12.5 μM to produce the calibration curve. For the study of antioxidant activity, the ABTS^•+^ solution was diluted with distilled water to reach an absorbance value of 0.70 (± 0.02) at 734 nm. Then, 10 μL of each sample and/or standard was pipetted in triplicate into wells of a 96-well microtiter plate (Nunc™ MicroWell™, ThermoFisher Scientific™), together with 190 μL of diluted ABTS solution, incubated for 6 min at room temperature in the dark, and read at 734 nm using a spectrophotometer (Epoch Biotek, Agilent Technologies Inc., Santa Clara, CA, USA). The results were expressed as μM Trolox.

### 2.10 Statistical analysis

The data results were expressed as means ± SD. STATA statistical software (StataCorp, College Station, TX, USA), version 16 (StataCorp, 2016) was used as statistical software to analyze the data. Previously, the normality of the data distribution was verified using the Shapiro–Wilk test. A one-way repeated-measures MANOVA test followed by Duncan’s *post hoc* test was performed to assess the significance of the treatment (*TRT*), time (*t*), and their combination effect (*TRT+t*) at each time point of the trial. The first of the two estimated models included the effects of *TRT* and *t*, and the second included their combination effect, to avoid multicollinearity issues. The sphericity assumption, which affects only ANOVA and not MANOVA, was not tested (36). Furthermore, to check for the robustness of our results, mixed models were also used, with group and time, or their interaction, as fixed effects and cage as random effects; the results were largely similar to the MANOVA ones and were omitted for brevity. In cases where the assumption of normal data distribution was not met, the non-parametric Kruskal–Wallis test was performed. For the combination effect (*TRT+t*), in addition to reporting the *p*-value for each experimental time point, the statistical significance of *TRT+t* effects was indicated using a superscript letter (^a, b^) at each time point. The statistical significance was set at *p* < 0.05 for all tests applied.

## 3 Results

### 3.1 Serum biochemistry

#### 3.1.1 Serum protein fraction

The serum TP, Alb, and Glb levels were not influenced directly by the *TRT* (*p* > 0.05) (Table 2). However, they were affected by *t* (*p* < 0.05), unlike the serum Glb content (*p* > 0.05). In particular, both serum TP and Alb levels increased in both groups at T3, compared to those found at T0 and T5 (*p* < 0.05). At the end of the trial, their levels returned to values similar to those detected at the start of the trial (*p* > 0.05) (Table 2). Additionally, at each time point (T0, T3, and T5), no differences between the control and treated group were found regarding the influence of the *TRT+t* combination effects on serum TP, Alb, and Glb content (*p* > 0.05) (Table 2).

**Table 2:** Effects of PHYTO-LAYER™ administration on the serum biochemical indices at the 41^st^ week of age (T0), 44^th^ week (T3), and at the end of the trial (T5).

Item	Time point						*p*-value*						
	T0		T3		T5		*TRT* ^!^	*t* ^$^			*TRT+t* ^♦^		
	Control	Treated	Control	Treated	Control	Treated		T0 vs. T3	T0 vs. T5	T3 vs. T5	T0	T3	T5
TP (g/L)	54.36 ± 4.31	54.92 ± 5.17	57.60 ± 5.05	57.36 ± 3.82	54.63 ± 5.66	53.30 ± 4.08	0.752	<0.032	0.608	<0.011	0.778	0.898	0.504
Alb (g/L)	21.44 ± 1.34	21.66 ± 1.46	22.60 ± 1.59	23.10 ± 1.72	21.03 ± 1.32	21.03 ± 1.37	0.468	<0.002	0.204	<0.000	0.701	0.391	1.000
Glb (g/L)	32.95 ± 3.14	32.42 ± 3.08	35.00 ± 3.77	34.18 ± 2.28	33.97 ± 4.81	32.27 ± 3.14	0.196	0.063	0.646	0.127	0.708	0.550	0.261
Alb/Glob	0.65 ± 0.04	0.65 ± 0.05	0.64 ± 0.05	0.67 ± 0.03	0.62 ± 0.05	0.65 ± 0.05	0.079	0.663	0.267	0.146	0.858	0.213	0.190
Ca (mmol/L)	6.96 ± 0.54	7.49 ± 0.78	7.89 ± 0.68	7.62 ± 0.69	7.58 ± 0.73	7.47 ± 0.71	0.767	<0.011	0.127	0.242	0.072	0.328	0.700
P (mmol/L)	2.14 ± 0.30	2.21 ± 0.37	2.53 ± 0.30	2.43 ± 0.29	2.26 ± 0.46	2.07 ± 0.26	0.333	<0.002	0.926	<0.002	0.606	0.457	0.190
Mg (mmol/L)	1.53 ± 0.10	1.60 ± 0.10	1.64 ± 0.09	1.66 ± 0.11	1.61 ± 0.14	1.63 ± 0.09	0.147	<0.014	0.077	0.413	0.085	0.654	0.743

TP, total protein; Alb, albumin; Glb, globulin; Ca, calcium; P, phosphorus; Mg, magnesium.

Values are means ± SD of 13 hen serum samples for each treatment group.

^!^*TRT*, treatment effect.

^$^ *t*, time effect.

^♦^*TRT+t*, treatment and time combination effects.

^*^The differences were considered significant for the *TRT* and *t* at *p* < 0.05.

### 3.2 Serum mineral content

The Ca, P, and Mg serum concentrations were not affected by the *TRT* (*p* > 0.05). However, their levels showed significant effects of *t* only at T3, compared to the values found at T0, with a slight increase in their content (*p* < 0.05) (Table 2). Furthermore, the P serum content was affected by the *t* effect also at the end of the trial (T5), where the treated group showed a lower level of serum P than that found at T3 (*p* < 0.05), albeit within the normal physiological range (Table 2). Overall, no significant differences were observed due to the *TRT+t* combination effect during the trial (*p* > 0.05) between the control and treated hens.

### 3.3 Egg-quality indices

#### 3.3.1 Egg weight

The egg weight of treated hens was positively influenced by the *TRT* (*p* < 0.05) (Table 3), with a progressive increase in weight observed from T0, after the *TRT*. During the trial, the egg weight of the control group decreased and remained consistently lower than that of the treated hens. At T3 and T5, the eggs produced by the treated hens were heavier than those of the control group (*p* < 0.05) (Table 3), and statistically significant *TRT+t* combination effects were detected, confirming the positive influence of the *TRT* in improving egg weight.

**Table 3:** Effects of PHYTO-LAYER™ administration on egg-quality indices at the 41^st^ week of age (T0), 44^th^ week (T3), and at the end of the trial (T5).

Item	Time point						*p-*value*						
	T0		T3		T5		*TRT* ^!^	*t* ^$^			*TRT+t* ^♦^		
	Control	Treated	Control	Treated	Control	Treated		T0 vs. T3	T0 vs. T5	T3 vs. T5	T0	T3	T5
Egg weight (g)	65.13 ± 2.28	64.76 ± 3.31	60.53 ± 3.74^a^	67.00 ± 4.19^b^	62.66 ± 3.25^a^	67.56 ± 2.99^b^	<0.000	0.069	0.802	<0.050	0.672	<0.000	<0.000
Yolk (g)	18.73 ± 1.22	18.62 ± 1.32	16.10 ± 1.86^a^	18.40 ± 1.83^b^	17.33 ± 1.56^a^	19.16 ± 1.34^b^	<0.000	<0.000	0.142	<0.000	0.780	<0.000	<0.000
Albumen (g)	36.56 ± 2.40	36.68 ± 2.77	36.06 ± 3.50^a^	39.86 ± 3.74^b^	36.63 ± 2.97^a^	39.06 ± 3.48^b^	<0.000	<0.037	<0.045	0.845	0.890	<0.000	<0.005
Shell (g)	9.83 ± 0.83	9.55 ± 1.05	8.36 ± 1.27	8.66 ± 1.21	8.70 ± 0.79^a^	9.33 ± 1.06^b^	0.168	<0.000	<0.001	<0.011	0.306	0.272	<0.021
Thickness (mm)	0.52 ± 0.07	0.45 ± 0.04	0.50 ± 0.05^a^	0.64 ± 0.07^b^	0.35 ± 0.05^a^	0.59 ± 0.05^b^	<0.000	<0.000	0.292	<0.000	0.067	<0.000	<0.000
Strength (N)	52.00 ± 10.1	53.14 ± 11.1	55.71 ± 6.86	53.67 ± 10.96	53.86 ± 7.76	53.08 ± 7.89	0.679	0.230	0.587	0.460	0.653	0.417	0.768
HU	91.21 ± 7.15	88.58 ± 6.64	92.09 ± 6.37^a^	84.05 ± 11.41^b^	94.05 ± 4.19^a^	89.58 ± 4.57^b^	<0.000	0.267	0.080	<0.006	0.207	<0.000	<0.046
Roche scale	15.02 ± 0.48	15.06 ± 0.44	15.66 ± 0.66	15.86 ± 0.34	15.13 ± 0.50	15.26 ± 0.58	0.388	<0.000	0.481	<0.000	0.349	0.134	0.349

HU, Haugh unit, calculated as: HU = 100 * Log10 (h – 1.7w 0.37 + 7.6), where: h = observed height of the albumen in millimeters (mm); w = weight of egg in grams (g).

Values are means ± SD of 60 eggs for each treatment group, sampled and analyzed at each time point.

^!^*TRT*, treatment effect.

^$^ *t*, time effect.

^♦^*TRT+t*, treatment and time combination effects.

^*^The differences were considered significant for the *TRT* and *t* at *p* < 0.05.

^a, b^ Means within a row with different superscript letters are significantly different at *p* < 0.05 for the *TRT+t* combination effect.

#### 3.3.2 Egg yolk and albumen weight

Following the improvement in egg weight, the *TRT* positively influenced the yolk and albumen weight (*p* < 0.05), with higher weights in eggs from treated hens than in the control group (Table 3). For the *t* effects, at T3 and T5, the eggs from the treated group had higher yolk and albumen weights, with significant *TRT+t* effects detected (*p* < 0.05).

#### 3.3.3 Eggshell weight, strength, and thickness

The *TRT* did not influence eggshell weight and strength (*p* > 0.05), although eggshell thickness was positively influenced in the treated group (*p* < 0.05) (Table 3). An increasing trend in the eggshell weight and thickness was also noted at different time points. Eggshell weight decreased from T0 to T3 and increased from T3 to T5 due to the *t* effects but remained higher in the treated group (*p* < 0.05). For the *t* effects, eggshell thickness increased from T0 to T3, with higher values in the treated group (*p* < 0.05). Eggshell strength was not influenced by the *t* effects (*p* > 0.05). The eggshell weight at T5 showed significant differences for the *TRT+t* combination effects (*p* < 0.05), while the eggshell thickness at T3 and T5 was higher in the treated group (*p* < 0.05).

#### 3.3.4 Haugh unit (HU) and Roche scale color

The HU was negatively influenced by the *TRT* (*p* < 0.05), as the treated hens produced eggs with a lower HU during the trial (Table 3). Owing to *t* effects, at T3, after the first phytoextract administration, the HU decreased in treated hens’ eggs from T0. However, at T5, after the latest treatment, HU increased in both groups but remained lower in treated hens’ eggs than in the control (*p* < 0.05). Differences were also detected for the *TRT+t* effects at T3 and T5, where eggs from the treated group had a lower HU than the control group (*p* < 0.05), confirming the potential negative effect of the TRT on this parameter. The yolk color intensity was not influenced by the *TRT* nor *TRT+t* effects (*p* < 0.05) (Table 3). Minimal but significant variations in color intensity were detected due to the *t* effect at T3 and T5 (*p* < 0.05). The treated group produced eggs with a slight increase in color intensity at T3 from T0; at the end of the trial, the intensity decreased in both groups but remained higher in the treated hens’ eggs.

### 3.4 Lipid oxidation of egg yolks

The MDA content in yolk samples was not influenced by the *TRT* (*p* > 0.05), with no effect detected for the t and *TRT+t* combination effects (*p* > 0.05) (Table 4). Lipid oxidation values remained constant in both hen groups, although at the end of the trial (T5), the MDA content of eggs from treated hens was numerically higher than in the control group, albeit not statistically significant (*p* > 0.05).

**Table 4:** Effects of PHYTO-LAYER™ administration on MDA content, total phenolic content, and antioxidant activity of egg yolks at the 41^st^ week of age (T0), 44^th^ week (T3), and at the end of the trial (T5).

Item	Time point						p-value*						
	T0		T3		T5		*TRT* ^!^	*t* ^$^			*TRT+t* ^♦^		
	Control	Treated	Control	Treated	Control	Treated		T0 vs. T3	T0 vs. T5	T3 vs. T5	T0	T3	T5
TBARS (μM MDA)	3.80 ± 1.45	3.94 ± 1.56	3.94 ± 2.12	3.64 ± 2.02	3.65 ± 1.88	4.19 ± 2.37	0.816	0.886	0.895	0.806	0.867	0.895	0.806
TPC (mg GAE/mL)	0.57 ± 0.08	0.59 ± 0.10	0.60 ± 0.08	0.72 ± 0.16	0.62 ± 0.12^a^	0.81 ± 0.06^b^	<0.026	0.363	0.121	<0.026	0.909	0.080	<0.041
ABTS (μM Trolox)	176.91 ± 39.80	178.66 ± 24.98	152.67 ± 42.99^a^	183.53 ± 23.46^b^	178.61 ± 47.40^a^	210.25 ± 20.56^b^	<0.024	0.363	0.121	<0.026	0.909	<0.047	<0.038

TBARS: thiobarbituric acid-reactive substances; TPC: total phenolic content; ABTS: 2′-azinobis-(3-ethylbenzothiazoline-6-sulfonic acid).

Values are means ± SD of 10 eggs for each treatment group, sampled and analyzed at each time point.

^!^*TRT*, treatment effect.

^$^ *t*, time effect.

^♦^*TRT+t*, treatment and time combination effects.

^*^The differences were considered significant for the *TRT* and *t* at *p* < 0.05.

^a, b^ Means within a row with different superscript letters are significantly different at *p* < 0.05 for the *TRT+t* combination effect.

### 3.5 Total phenolic content (TPC) and antioxidant activity of egg yolks (ABTS)

The TPC of the yolk samples, expressed as mg GAE/mL, and ABTS (mM Trolox) are shown in Table 4. The TPC was positively influenced by the *TRT*, as the egg yolks of treated hens showed higher values than the control group (*p* < 0.05). This parameter was also influenced by the *t* effect: at the end of the trial (T5) in particular, the TPC value was higher in the treated group than in the control hens’ eggs (0.81 ± 0.06 mg GAE/mL in the treated group, 0.62 ± 0.12 mg GAE/mL in the control group). From T0, the TPC content increased in the treated hens (T0, 0.59 ± 0.08 mg GAE/mL to T5, 0.81 ± 0.06 mg GAE/mL) during the trial, relative to the control. A trend was also observed for the *TRT+t* combination effect, supporting the positive effect of the treatment in increasing the total polyphenol content (*p* > 0.05).

The *TRT* positively influenced the antioxidant activity (ABTS assay) of eggs from the treated hens (*p* < 0.05), which showed higher values than the control groups, from the average value of 178.66 μM Trolox at T0 to 210.25 μM Trolox at T5. Concerning the t effects, the antioxidant capacity significantly increased from T3 to T5 in eggs of treated hens (*p* < 0.05). It can be observed that the antioxidant capacity of eggs from the control group decreased at T3 compared to T0 and remained consistently lower than in the treated hens’ eggs throughout the trial. Overall, no significant differences were observed due to the *TRT+t* during the trial (*p* > 0.05) between the control and treated hens.

## 4 Discussion

Over the past 30 years, global egg production from caged-laying hens has remarkably increased (14). High productivity and efficiency characterizing current laying hens’ genetic lines can increase the risk of animals experiencing health and welfare problems due to high metabolic stress (37). Given the increasing consumer demand for natural and healthy feed additives to support animal health and nutrition, dietary plant-derived supplements with antioxidant and anti-inflammatory properties have been proposed as potential preventive measures (38).

This study investigated the effect of supplementation with a standardized extract of SIL + CGA association to maintain and improve egg quality, including lipid oxidation, antioxidant capacity, and some serum indices, in caged-laying hens affected by liver disorder (26). High egg production can be associated with significant alterations in blood indices, related to protein metabolism (39). Serum protein fractions commonly used in nutritional studies to assess body condition in birds (40, 41), such as TP, Alb, and Glb, were not negatively influenced by the SIL + CGA intermittent supplementation in this study. The values observed remained within the physiological range throughout the trial (42–44), confirming no alteration of the protein metabolism. Serum minerals evaluated, such as Ca, P, and Mg, are most important in the diet of laying hens, to support egg production and egg quality, skeletal structure, and metabolism (45, 46). In this study, the serum content of Ca, P, and Mg was not affected by phytoextracts administration, although minimal physiological variations over time were detected. However, the serum concentrations were higher than those reported by other studies investigating the same parameters on hens (47, 48). This may demonstrate that despite liver metabolic disorders previously diagnosed (26), the treated hens could maintain good levels of these minerals within optimal ranges in the bloodstream, to support egg production and their physiological functions.

Egg quality directly impacts the efficiency, productivity, and overall health of laying hens (49). The oxidative stress that increases in laying hens during late egg production can be responsible for the decrease in egg quality (50, 51). In this study, SIL + CGA supplementation, administered intermittently, exerted a significant positive effect on various key egg-quality indices. Egg weight, a direct proportion of albumen, yolk, and shell, is an important trait that influences egg quality and grading (52). After the latest phytoextract administration, egg weight was significantly improved in treated hens compared to the control group, especially at the end of the trial. In this regard, preliminary results on liver functionality revealed that ALT enzyme activity in treated hens was significantly reduced. Contrarily, in control hens, a medium and severe liver steatosis was confirmed by histological evaluation (26); it cannot be excluded that the liver injury negatively affected the egg weight in control hens, compared to the positive effects of phytoextracts in the treated hens, which reduced the liver damage, allowing a good maintenance of egg weight. It has been previously reported that dietary silymarin supplementation improves egg quality and egg weight. The study conducted by Faryadi and colleagues (53) reported, in fact, that laying hens supplemented with 100 and 200 mg/kg BW of silymarin produced heavier eggs than the control group. The same positive effect was observed in this trial with a lower dose of SIL administered by hens (~0.44 mg/day, considering that each treated hen assumed a mean daily water intake of ~200 mL; data not shown), probably enhanced due to a synergistic effect with CGA. This result, associated with a proportional increase in the yolk and albumen weight as observed in this trial, certainly represents a positive aspect, especially from a commercial point of view, representing a better choice for consumers.

Ensuring eggshell quality is crucial for the egg industry, and eggshell thickness is considered one of the main indirect measures of eggshell quality. Despite no variation in serum mineral content in the analyzed eggs, the eggshell thickness increased with the SIL + CGA treatment, without affecting eggshell strength and eggshell weight. This aligns with previous studies testing the same phytoextracts, although administered separately (17, 54, 55), showing effective results of their association potential. However, differences and controversial results in eggshell thickness and its strength are sometimes reported. The study conducted by Tatara and colleagues (56) in quails revealed a negative correlation between eggshell thickness and its strength, indicating that the mineral content and the spatial microarchitectural arrangement may influence the eggshell strength and its thickness. Even if the thickness is the main factor that supports the eggshell strength, thicker eggshells do not ensure stiffer or stronger shells (57). In cases like this, where shell thickness increased but breaking strength was not affected, the explanation lies in the shell ultrastructure’s good construction, including the distribution of calcite crystals (58). These findings agree with our previous study conducted on laying hens supplemented with a commercial standardized mixture of bioactive compounds, although no modifications of the strength were evidenced (19). Indeed, the authors hypothesized that this event was probably due to the non-optimal arrangement of calcite crystals during eggshell development, also in line with the results reported by Obianwuna and colleagues (59); they reported no positive effect on breakage resistance of eggshell, but only on thickness. Thus, it cannot be excluded that phytogenic supplementation may improve mineral deposition without necessarily altering shell architecture. However, given the presence of conflicting results among the different studies, particularly concerning the ultrastructural evaluation of shells, further investigations would be warranted to better clarify the potential influence of feed additives on this quality parameter, which is important for commercial purposes.

The Haugh unit (HU) represents one of the most important indices of albumen quality. This parameter is influenced primarily by the ovomucin content of the egg, not only by egg freshness (60). In our experiment, the eggs were collected and evaluated simultaneously, so the differences in HU between the treated and control groups were not influenced by variations in egg freshness. A worse HU was observed in eggs produced by treated hens after the first SIL + CGA treatment, and at the end of the trial, compared to the beginning of the trial, while in the control group, these values remained constant. Although the phytoextract administered reduced HU, its values were still in the range of 84–89, which aligns with data in the literature for this hen hybrid (61). However, another hypothesis can be correlated to the serum MDA content (albeit not significant) found in treated hens. Preliminary data, in the same hens, showed a slight numerical increase in serum MDA in treated hens (T0: treated: 35.71 ± 7.70 μmol/L; control: 36.54 ± 24.01 μmol/L; T5: treated: 44.15 ± 17.89 μmol/L; control: 33.41 ± 7.34 μmol/L). This relatively negative result is probably due to greater or partial metabolisation of fats. Although the hepatoprotective effect of SIL + CGA (26) remains difficult to explain, it cannot be excluded that it also influenced the HU. This hypothesis would deserve further investigation, including the evaluation of albumen protein (e.g., ovomucin), viscosity, or the albumen MDA content.

Yolk color, primarily influenced by dietary carotenoid intake, is a key indicator of egg quality, with darker yolks generally preferred by consumers (62). In this study, treated hens showed a slight but non-significant increase in yolk color. Carotenoids from plant-based diets are transferred to the yolk and absorbed efficiently in the small intestine. Owing to its antioxidant properties, silymarin may help accumulate carotenoids in the yolk and protect them from oxidative damage (63). Previous studies have also shown that these extracts can enhance yolk pigmentation. An increase in yolk color has been observed following dietary supplementation with 0.03 and 0.06% milk thistle in Hy-Line Brown hens (1), as well as in the study by Radwan and colleagues (54), where quails fed different levels of artichoke leaf meal showed an enhancement in yolk color, likely due to the carotenoid content of the extract.

In recent years, researchers have paid increasing attention to the antioxidant capacity of eggs because antioxidants may play an important role in defending against chronic diseases (64). Supplementation with botanical extracts can increase the natural presence of antioxidants in eggs and contribute to the lipid oxidative stability of the yolk and albumen (65). Egg yolk is rich in lipids, mainly phospholipids, which, due to the greater unsaturation of fatty acids, are particularly susceptible to oxidation during processing and storage (21). The TBARS assay was employed to determine lipid peroxidation in eggs. It was found that the SIL + CGA administration did not influence the yolks’ MDA content, an end-product of lipid oxidation, which represents a key indicator of oxidative stress within biological systems (66). It has been reported, however, that egg yolk MDA concentration normally increases during storage (53, 67). In this study, the MDA concentration remained constant in eggs from both experimental groups. The MDA level in eggs is directly correlated to the yolk’s lipid composition and the antioxidants deposited in the eggs (68, 69). The antioxidant effect of phenolic compounds is due to their ability to counteract free radical production, chelate metal ions, and inhibit or reduce the formation of singlet oxygen. However, the metabolism and bioavailability of polyphenols have some limitations, as some are poorly absorbed in the small intestine and require enzymatic hydrolysis by intestinal microbiota. The rapid absorption and excretion of polyphenols (natural antioxidants) is a limiting factor that determines their low accumulation in tissues compared to synthetic antioxidants (65). The lack of alteration in egg yolks’ MDA content, as observed in this study, could indicate a lower deposition of plant-derived antioxidants than would occur using synthetic ones (e.g., butylated hydroxytoluene, butylated hydroxyanisole, and vitamins) (70). Therefore, the increase in antioxidant capacity observed in the egg yolks may be due to greater absorption of pigments from the diet, supported by the phytoextract administered. Phenolic compounds, the most abundant secondary metabolites in plants, exhibit numerous well-documented biological activities (71–73). Owing to the ability of hens to transfer health-promoting components (e.g., vitamins, minerals, and polyphenols), dietary supplementation could be a successful strategy to improve the nutraceutical value of eggs (74). Some studies present in the literature have investigated the bioaccumulation of antioxidant compounds into eggs (75–77), including CGA (78). The TPC measured by the Folin–Ciocalteu assay increased in egg yolks of treated hens, indicating an improved antioxidant capacity and, consequently, egg quality. Indeed, the antioxidant properties of these two botanical species can be associated with the phenolic compounds they contain, namely flavonoids such as silymarin, cynarin, chlorogenic acid, apigenin and luteolin in artichoke (45). In line with our observations, similar results were obtained by several studies in terms of an increase in TPC in egg yolk, although not with the same phytoextracts (79–81). The treatment significantly increased the egg’s antioxidant capacity as evidenced by elevated ABTS value in yolk samples, indicating an amelioration of the storage potential, providing a basis for further studies on the possible improvement of nutraceutical properties.

The ABTS radical scavenging capacity depends on antioxidant compounds present in the sample; eggs are known to contain several natural antioxidant compounds, including proteins, peptides, carotenoids, tryptophan, tyrosine, and polyphenolic compounds (64). As the nutritional content of eggs is related to the diet of the laying hens, eggs rich in antioxidant compounds can be obtained by adjusting the hens’ diet. The amount of phenolic compounds in the egg yolk increased over time, as observed by the Folin–Ciocalteu test results, with a consequent increase in antioxidant activity, verified by the ABTS assay, as observed at the end of the trial. These effects can be attributed to the important role of bioactive compounds administered. At the same time, the production of eggs with enhanced nutritional value is a desirable condition from the consumer’s point of view. We can hypothesize that the antioxidant effect of the supplemented phytogenic extracts does not necessarily increase yolk pigmentation: not all polyphenols possess pigmenting capacity, and yolk colouration is most likely driven primarily by lipophilic carotenoids (82). As it is known that the mechanism of antioxidant deposition in eggs is influenced by both lipid metabolism and the age of the hens, we can affirm that our results are encouraging, as they show that the phytoextracts supplemented were effective in counteracting the physiological declines mentioned above. This finding is of great importance, as this is the first *in vivo* study that has evaluated the antioxidant capacity of egg yolk after SIL + CGA association extract supplementation in laying hens, to the best of our knowledge.

## 5 Conclusion

This study demonstrates that the intermittent administration of 1 mL/L SIL + CGA in caged-laying hens, previously shown to undergo high hepatic metabolic stress, was able to maintain and even improve several key egg-quality traits, including antioxidant capacity, beyond the peak of production. These findings are particularly relevant given the growing interest of both producers and consumers in egg quality, which directly contributes to the nutritional value of eggs. Nevertheless, further research is warranted to assess whether comparable changes in egg quality can be achieved under different supplementation protocols (e.g., continuous administration), varying doses, or across hens of different ages. Trials testing the individual phytocompounds would also provide valuable insights for a clearer comparison of their effects. In addition, such studies could clarify whether the administered bioactive additives or their metabolites persist in edible matrices (i.e., yolk and albumen) and whether they play a positive or negative role in modulating egg quality. Collectively, future data will help optimize the use of these botanical feed additives, promoting their flexible and sustainable application (e.g., considering phytoextract production costs) on a larger scale as effective natural tools to enhance both egg quality and nutraceutical potential.

## Data Availability

The raw data supporting the conclusions of this article will be made available by the authors, without undue reservation.
